# A Multi-Modal Mobile Phone-Based Communication Strategy to Maximize Retention in a Post-Intensive Care Follow-up Study of Acute Respiratory Distress Syndrome Survivors

**DOI:** 10.21203/rs.3.rs-9346873/v1

**Published:** 2026-05-14

**Authors:** Charity O. Ogunlusi, Ansley E. Jones, Margarita Mira-Sanchez, Arooj Fatima, Victor D. Dinglas, Dale M. Needham, Peter Scala, Richard Ellsworth, Matthew R. Baldwin

**Affiliations:** Columbia University; Columbia University; Columbia University; Johns Hopkins University; Johns Hopkins University; Johns Hopkins University; NewYork–Presbyterian Hospital; NewYork–Presbyterian Hospital; Columbia University

**Keywords:** post-intensive care syndrome, acute respiratory distress syndrome, survivors, engagement, retention, mobile health, cohort study

## Abstract

**Background:**

Retention of critical illness survivors in longitudinal studies is challenging but essential to minimizing selection bias. Contact via telephone and mail may be less effective in the era of text messaging using mobile phones. We designed, implemented, and evaluated mobile phone-based communication, rideshare coordination, and renumeration protocol to optimize retention among acute respiratory distress syndrome (ARDS) survivors.

**Methods:**

We conducted a single-center cohort study of adult ARDS survivors, assessing disability via surveys at hospital discharge, and at 3-, 6-, and 12-months. We also conducted performance-based measures and phlebotomy at hospital discharge and 3-months. We sent mobile phone-based text message reminders 14 and 8–11 days prior to each follow-up due date. We then contacted participants 7–10 days before their due date using telephone calls, text messages, or email, with proxies contacted when necessary. We provided mobile phone- or email-based remuneration at hospital discharge and 3-month clinic visits. For this clinic visit, we coordinated transportation using a mobile phone rideshare application, including accessible options for participants with mobility limitations.

**Results:**

Participants were a median [IQR] 61 [43–69] years old; 32% were Black, 30% were Spanish-speaking, and 31% had less than high-school education; 20% had psychiatric comorbidity, and 19% had alcohol or drug misuse. Among 60 eligible survivors, we achieved 96–98% retention at 3, 6, and 12-month follow-up telephone visits, and 80% retention at 3-month clinic visits. Participants completed the 3-, 6-, and 12-month telephone surveys with a median [IQR] of 1 [−3 to 8], 3 [−3 to 17], and – 1 [−6 to 8] days of the due date, with a median [IQR] of 1 [1 to 2] telephone calls for each follow-up. Median telephone call attempts and assessment timing did not differ by age, language, or educational attainment (all p > 0.05). Complete core physical function data were obtained at all assessments.

**Conclusion:**

A mobile phone-based communication strategy achieved high follow-up rates with minimal contact attempts and timely completion of assessments across diverse demographic groups. Rideshare coordination, including accessible transportation options, facilitated in-person retention for 3 months. These methods demonstrate a scalable approach to maximizing retention in longitudinal studies of ICU survivors.

## BACKGROUND

Assessment of longitudinal patient outcomes after critical illness is essential for understanding and improving physical debilitation, cognitive impairment, or mental health morbidities, including depression, anxiety, and post-traumatic stress disorder, that comprise post-intensive care syndrome (PICS), and for evaluating the efficacy of novel interventions to treat it. High participant retention is fundamental to the validity of study findings. Retaining critical illness survivors in studies requiring multiple longitudinal assessments remains a substantial challenge for several reasons. Many ICU survivors have poor baseline health and often face new or worsened physical and neuro-psychological morbidities after hospitalization that may impair their ability or desire to respond to follow-up attempts or participate in either phone, video, or clinic-based assessments [1-4]. Conversely, those who recover successfully may return to work, limiting their time available to participate in research-related assessments [5]. Low retention rates can introduce selection bias, reduce statistical power, and threaten the internal validity of the study, particularly when systematic differences exist between participants retained and those lost to follow-up [6-8].

Systematic reviews of longitudinal studies have identified multiple strategies that improve retention and minimize attrition. Evidence supports the use of frequent, multifaceted contact attempts (e.g., mail and telephone calls) by highly trained research staff to enhance participant engagement [7]. Cohort studies achieving high retention typically employ highly skilled research personnel, culturally responsive and personalized outreach, and ongoing evaluation and refinement of retention practices [6, 9].

Despite these insights, important gaps remain in understanding which strategies are most effective in contemporary research settings, particularly in the era of telecommunication technological advancements. Many prior studies were conducted before the widespread adoption of mobile phone-based communication and “app” technologies. In addition, published protocols often underreport the adaptive and iterative nature of retention strategies, which are frequently modified during the study’s course in response to emerging challenges. Although multiple contact attempts using different modalities, such as phone calls, letters, and emails, conducted at varying times of day and on different days of the week have been shown to improve retention rates [7], traditional approaches, such as telephone calls and postal mail may be less effective in the current mobile phone-based telecommunication era, characterized by increased robocalls and telephone spam, declining voice communication, and an increased preference for text-based messaging.

In response to these challenges, we sought to design, implement, and perform a preliminary evaluation of a comprehensive participant retention protocol integrating mobile phone- and web-based “app” interventions for communication with additional logistical support mechanisms, including rideshare coordination and electronic participant remuneration to maximize retention. These strategies were specifically designed to optimize retention for both phone and in-person follow-up assessments in a diverse cohort of Acute Respiratory Distress Syndrome (ARDS) survivors in New York City and its suburbs.

## METHODS

### Study Design and Participants

We conducted a single-site study at Columbia University Irving Medical Center (CUIMC), New York, within an ongoing prospective longitudinal cohort study of ARDS survivors. Beginning in January 2023, we enrolled community-dwelling ambulatory adults aged ≥ 18 years who met the global definition for ARDS [10] and survived to hospital discharge at the tertiary-care Milstein or the community-level Allen Hospitals at CUIMC. We excluded those with pre-existing neuromotor disease, dementia, solid or bone-marrow transplant, ARDS due to trauma or autoimmune disease, and metastatic malignancy. Additional exclusion criteria are described in E-Table 1. Participants were enrolled during the week prior to hospital discharge, and were followed up by telephone at 3, 6, and 12 months after discharge. Participants were also invited to complete an in-person study clinic visit at 3 months. As of December 2025, 70 participants were enrolled. The first three participants completed their 3-month follow-up prior to implementation of the mobile health retention protocol and were therefore excluded from 3-month retention analyses. The study was approved by the Columbia University Institutional Review Board (AAAU6985). All subjects provided informed consent to participate in the study.

### Mobile Health Retention Modalities

**Figure 1:**
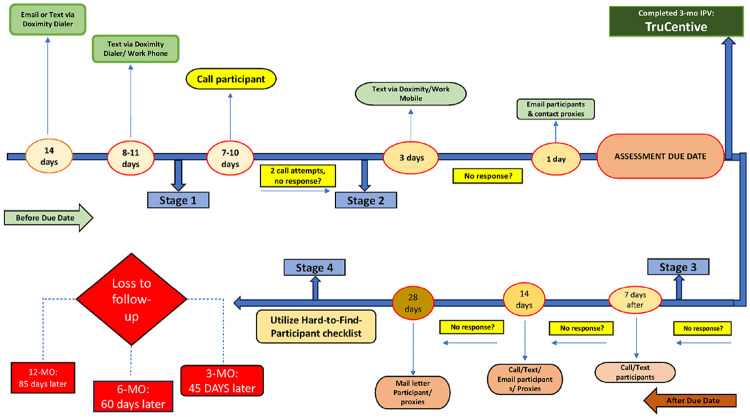
Time-based escalation framework for participant contact. The figure illustrates the prespecified, staged participant contact protocol used to facilitate retention for longitudinal follow-up assessments. Initial outreach began 14 days before each scheduled assessment due date using automated text messages and voicemail messages delivered via Doximity Dialer. Escalation to additional contact modalities, including telephone calls and text messages from designated study mobile phones, email correspondence, and proxy outreach, was triggered by non-response at predefined intervals before and after the due date. Participants who remained unreachable entered an extended follow-up workflow incorporating additional contact attempts and proxy outreach. Electronic remuneration was delivered following completion of follow-up assessments.

We implemented a multimodal mobile phone–based retention strategy designed to address common barriers to longitudinal follow-up among critical illness survivors, including scheduling challenges, communication preferences, work-hour constraints, mobility challenges, and delays in participant reimbursement (Fig. 1).

Web-based communication included automated text message reminders and voicemail outreach delivered via the Doximity Dialer^™^ app (San Francisco, CA, USA). We sent initial reminders 14 days before each due date to facilitate scheduling. We delivered voicemail messages using Doximity Dialer’s direct-to-voicemail functionality, allowing asynchronous contact without initiating live calls for participants unavailable during work hours. We sent an additional reminder 8–11 days before the due date using study mobile phones to reinforce follow-up expectations and increase message visibility.

We conducted direct participant contact using telephone calls and text messages from study mobile phones that research coordinators carried with them and checked daily. Use of study mobile phones ensured consistent caller identification, reduced the likelihood that study-related communication would be misidentified as spam, and supported bidirectional communication when rescheduling or clarification was needed. Moreover, this approach ensured research coordinators could respond promptly to study participants if they were away from their office phone or working from home without the need to use their personal mobile phone. We used email correspondence to supplement telephone and text-based outreach, particularly for appointment reminders, follow-up coordination, and reimbursement notifications. When participants were unreachable, we contacted previously designated proxy contacts in accordance with study consent procedures.

Participant compensation was provided electronically using the TruCentive^™^ platform (Palo Alto, California, USA) to minimize administrative burden, reduce delays, and minimize potential loss or theft associated with traditional cash, gift card, or check payment methods. Participants selected multiple reimbursement options, including cash-equivalent payments and electronic gift cards to major retailer businesses, with remuneration delivered via mobile phone or email based on participant preference.

For participants who consented to in-person follow-up visits, we coordinated transportation using the Lyft^™^ Business application (San Francisco, California, USA) to reduce transportation-related barriers. Lyft is a ride-sharing transportation network company that connects passengers with drivers through a mobile application. Lyft Access, a service that provides wheelchair-accessible vehicles and other accommodations for riders with mobility impairments, was used for participants requiring accessible transportation due to mobility limitations. We implemented all contact modalities within a prespecified, time-based escalation framework in which progressively intensified outreach was triggered by nonresponse at predefined intervals before and after each assessment due date. A schematic of the escalation protocol is shown in Fig. 1.

### Survey and Clinical Measurements

Survey-based assessments were administered during the week prior to hospital discharge, and at 3, 6, and 12-month follow-up visits. We conducted surveys of physical disability, including assessments of the basic and instrumental activities of daily living and Duke Activity Status Index [11-13], screened for depression and anxiety [14], post-traumatic stress disorder [15], and cognitive impairment, and assessed other measures of quality-of-life and symptoms (see E-Table 2 for details).

Performance-based assessments were conducted at baseline visit, at hospital discharge, and at a 3-month in-person visit, including hand grip dynamometry, six-minute walk distance (6MWD) [16], and the short physical performance battery (SPPB) [17]. Phlebotomy was conducted during the week prior to hospital discharge and at the 3-month in-person study clinic visit.

### Study Outcomes

Participant retention, the primary outcome, was defined as the total number of participants who completed the core physical function surveys by phone or during a 3-month in-person assessment, divided by the total number of participants who were eligible for follow-up at that same time, excluding those who died by the follow-up time point.

In secondary analyses, we examined the number of days between the due date and actual follow-up completion. Scheduled 3-month, 6-month, and 12-month follow-up time points were defined as 90, 180, and 365 days after the date of hospital discharge. The actual assessment date was defined as the date when the primary outcome physical function questions were completed by phone, or the date the participant agreed to present to our clinic for in-person completion of the questions. We also counted the number of telephone attempts prior to completing the core physical function questions or scheduling the in-person clinic visit, during which the surveys were completed.

### Statistical Analyses

We examined unadjusted between-group differences using the Mann-Whitney and chi-squared or Fisher’s exact test. We conducted analyses stratified by the median age of the study population, education level (less than high school versus high school or higher education), and language (Spanish vs. English). Analyses were performed using Stata V17 (Texas, USA).

## RESULTS

Among the first 65 participants enrolled, 5 died before the 3-month follow-up, 1 died between the 3- and 6-month follow-up, and 3 died between the 6- and 12-month follow-up (E-Figure 1). The median [IQR] age at hospital admission was 61 [43–69] years, and 60% were male. Nearly half the cohort was Hispanic (30% were Spanish-speaking only), and a third were Black. Nearly one-third did not graduate from high school. Multimorbidity was low (median [IQR] Charlson comorbidity score 1 [0–2]), with the prevalence of major cardiac, pulmonary, renal, or liver disease ranging from 3% to 9%. Nearly one in five had a psychiatric illness, most commonly depression or anxiety. Excess alcohol and illicit drug use were common (11% and 8%, respectively). Participants rarely had any baseline disability or clinical frailty (clinical frailty score > 5) prior to hospitalization (Table 1).

A total of 42 (65%) participants received invasive mechanical ventilation, and 23 (35%) received high-flow nasal oxygen or non-invasive mechanical ventilation support only, as is now part of the ARDS global definition criteria [10]. Most participants had moderate or severe ARDS and multi-organ failure while in the ICU, with 16 (25%) requiring extracorporeal membrane oxygenation (ECMO) support [18]. On the first day of ARDS, the median [IQR] PaO2/FiO2 ratio was 91 [70–150], and the median [IQR] SOFA was 11 [9-13]. At hospital discharge, half of the participants were too weak to participate in the 6WMD assessment (percent-predicted-6MWD median [IQR] 0% [0–37%]). The median [IQR] SPPB score was 3 [0–7], indicating that at least half of the participants needed assistance standing up [17]. Only 32 (49%) of participants were discharged to home, with the other participants discharged to post-acute inpatient facilities (Table 2).

There were 60 participants eligible for 3-month follow-up, of whom 48 (80%) consented to in-person 3-month clinic follow-up, 55 participants eligible for 6-month follow-up, and 32 participants eligible for 12-month follow-up, with 28 participants not yet in the 12-month follow-up window. At 3-month follow-up, there was 98% retention (one participant answered the phone but declined to answer questions) with completion of survey questions via telephone occurring a median of 1 [−3 to 8] day within the 3-month due date. The 3-month clinic follow-up occurred a median of 8 [0 to 41] days within the 3-month due date, in part due to scheduling a time when the patient and clinic space were both available. Retention at 6- and 12-month follow-up was 96% and 97%, respectively, with telephone follow-up of survivors usually occurring during the scheduled week of follow-up (Table 3). Among participants eligible for 6-month follow-up, one answered the telephone but declined to complete the survey, and one was unable to participate due to severe debility and the absence of any surrogate. Among participants eligible for 12-month follow-up, one answered telephone calls but declined to complete the survey.

In stratified analyses, at 3, 6, and 12-month follow-up, we observed similar median call attempts and success with scheduling assessments between younger and older participants (defined by the study sample median age of 61 years), English vs Spanish-speaking participants, and those with less than or greater than a high school level of education (all p > 0.05). However, we note that the upper quartile of each of the subgroups of older, Spanish-speaking, and higher education participants had 6-month telephone assessments done nearly 1 month after the 6-month post-discharge date (Table 4).

## DISCUSSION

Longitudinal follow-up of survivors of critical illness remains a persistent challenge, particularly among populations with substantial physical debility, psychiatric comorbidity, language barriers, and socioeconomic disadvantages. In an ongoing prospective cohort study of survivors of ARDS critical illness, consisting of many participants with a history of psychiatric illness and drug and alcohol misuse, we successfully implemented a mobile phone-based strategy that combined multimodal communication, rideshare coordination for clinic visits, and flexible electronic remuneration. Despite enrolling a demographically diverse cohort, including a high proportion of Spanish-speaking only participants and individuals with limited educational attainment, we achieved consistently high retention at 3, 6, and 12 months with timely completion of follow-up assessments.

Retention at all timepoints was ≥ 96%, exceeding retention reported in many longitudinal ICU survivorship studies, where pooled follow-up rates at similar intervals have ranged from approximately 75–81% [8, 19]. While pooled retention estimates specific to ARDS survivors are limited, longitudinal studies of acute respiratory failure and ARDS survivors have usually reported lower and more variable follow-up completeness, reflecting substantial heterogeneity in study design and retention practices [8]. Although we conducted our study at a single urban center, our findings suggest that mobile phone-enabled retention strategies may be feasible and acceptable for contemporary longitudinal studies of ICU survivors, particularly as responsiveness to traditional voice calls declines and text-based communication is increasingly preferred [20, 21].

Prior ICU survivorship studies have demonstrated that high retention rates (> 80%) can be achieved through resource-intensive protocols relying on repeated telephone contact, mailed correspondence, and highly trained research staff [6, 7]. While our retention rates were comparable, our protocol required fewer contact attempts and shorter lead times for scheduling. This difference likely reflects shifts in communication norms over the past decade, with declining responsiveness to voice calls and greater immediacy of text-based communication. Systematic reviews of retention strategies similarly emphasize that persistence alone is insufficient; rather, alignment with participant communication preferences and reduction of participant burden appear more influential for sustained engagement [6].

Notably, prior work in ARDS survivors has identified participant-level factors associated with incomplete longitudinal assessments, but few have examined protocol-level strategies to mitigate these challenges [4, 22]. A recent scoping of ICU survivorship research review found inconsistent reporting of retention methods and emphasized the need for clearer guidance and use of best practices to improve study design and follow-up completeness [23]. By explicitly defining, implementing, and evaluating a prespecified, multimodal retention protocol, our study directly responds to these methodological gaps.

Several methodological features may have contributed to the early success of our protocol. Participant contact was structured within a prespecified, time-based escalation framework, enhancing reproducibility and transparency, allowing outreach intensity to be systematically increased only when participants do not respond. Asynchronous communication modalities, including text messaging and direct-to-voicemail outreach, enabled engagement outside standard work hours and may explain high responsiveness with fewer live contact attempts. These methods were inspired by the review and modernization of cohort retention methods first described by the National Institutes of Health-funded Improving Long-Term Outcomes Research for Respiratory Failure initiative[24]. In addition, rideshare coordination with accessible transportation addressed a major structural barrier to in-person follow-up among physically debilitated survivors, where the majority of participants lived within a 30-minute drive of the medical center, contributing to high in-person retention at our 3-month study clinic visit. Flexible electronic remuneration further reduced administrative burden and supported continued engagement.

Our findings contribute to a growing body of literature emphasizing the importance of tailored contact strategies in maintaining longitudinal follow-up. Systematic evaluations of retention practices have identified reminders, flexible communication, and barrier-reduction strategies as commonly associated with high retention rates [6]. Studies with ≥ 80% retention over one year have highlighted that sustained, persistent, and differentiated approaches tailored to individual participant needs promote engagement [8]. However, prior reviews also caution that employing a greater number of strategies per se does not guarantee improved retention; rather, approaches that reduce participant burden and enhance accessibility appear most impactful [25].

While our modest sample size may limit our ability to detect differences between subgroups, retention did not appear to differ by age, language preference, or educational attainment, suggesting that combining barrier-reduction strategies with language-concordant and flexible outreach may mitigate disparities in longitudinal follow-up. This equity in retention represents an important methodological advance, as differential attrition by language or socioeconomic status can introduce bias and threaten internal validity. Nevertheless, some variability in timing, particularly among older, Spanish-speaking, and higher-education groups, warrants further investigation into contextual factors influencing engagement.

Our study has limitations. The protocol was evaluated at a single center and with a modest sample size, which may limit generalizability and precision, particularly to rural or resource-limited settings. However, mobile-phone-based technology exists in many resource-limited settings and is currently being evaluated to improve adherence to tuberculosis treatment [26]. The multimodal nature of the retention protocol precluded assessment of the relative contribution of individual components, but the low median number of telephone call attempts to obtain follow-up data reflects the importance of initiating contact with text messaging. Future studies should prospectively evaluate participant-centered outcomes, cost-effectiveness, and scalability of multimodal retention strategies in larger, multicenter cohorts, while carefully avoiding financial coercion.

## CONCLUSION

We designed and implemented a mobile phone-based participant retention protocol in a racially, ethnically, and socioeconomically diverse cohort of ARDS survivors in New York City, achieving high longitudinal follow-up with fewer contact attempts compared to prior reports. This structured, participant-centered protocol may inform future studies of critical illness survivorship and other longitudinal cohorts involving populations with substantial debility and multimorbidity.

## Supplementary Material

This is a list of supplementary files associated with this preprint. Click to download.

• BMCHSResearchSupplementR1.docx

## Figures and Tables

**Table 1:** ARDS survivor baseline characteristics

Demographics	n = 65
Age, years, median [IQR]	61 [43–69]
Male sex, n (%)	39 (60)
Hispanic, n (%)	29 (45)
Race, n (%)	
White	40 (62)
Black	21 (32)
Other	4 (6)
Primary language English	39 (70)
Education Level	
<High school education	20(31)
High school education	15(23)
College and/or graduate education	30(46)
**Comorbidities**	
Charlson comorbidity score, median [IQR]	1 [0–2]
COPD, n (%)	6 (9)
ILD, n (%)	2 (3)
CHF, n (%)	5 (8)
Liver disease, n (%)	2 (3)
Renal Disease, n(%)	5 (8)
Psychiatric Disease and/or Treatment	13 (20)
Depression, n (%)	9 (14)
Anxiety, n (%)	5(8)
Other, n (%)	2(3)
Excess alcohol use history	7(11)
Illicit drug use, during the month prior to admission	5(8)
**Pre-hospitalization physical function based on self-report**	
Clinical Frailty Scale, Median [IQR]	3 [2–4]
ADL Dependencies, Median [IQR]	0 [0–0]
IADL Dependencies, Median [IQR]	0 [1 – 0]

* ADL: activities of daily living; IADL: instrumental activities of daily living; COPD: chronic obstructive pulmonary disease; ILD: interstitial lung disease; CHF: congestive heart failure.

**Table 2:** ARDS survivor critical illness and hospital discharge clinical characteristics

Critical illness characteristics	n = 65
Ventilator Support	
Invasive mechanical ventilation, n (%)	42 (65)
High-flow nasal oxygen or non-invasive positive pressure ventilation only, n (%)	23 (35)
ARDS day 1 PaO2/FiO2 ratio, median [IQR]	91[70–150]
ARDS day 1 SOFA score, median [IQR]	11 [9–13]
Ventilator days, median [IQR]	11 [6–26]
ECMO, n (%)	16 (25)
Hospital length of stay, days, median [IQR]	20 [11–46]
**Physical function at hospital discharge**	
6-minute walk distance, meters, median [IQR]	0 [0-214]
%-predicted 6-minute walk distance, median [IQR]	0 [0–37]
SPPB, median [IQR]	3 [0–7]
Hand-grip strength, kg, median [IQR]	16 [10–25]
Weak hand grip*, n (%)	51 (78)
**Discharge Location**	
Sub-acute rehabilitation, long-term acute care	24 (37)
Acute rehabilitation	7 (11)
Home	32 (49)
Other**	2 (3)

SOFA: Sequential Organ Failure Assessment. SPPB: short physical performance battery.

* Weak grip based on Fried physical frailty criterion.

** Other: 1 home with custodial help, 1 inpatient drug rehabilitation. ECMO: Extracorporeal Membrane Oxygenation.

**Table 3:** Retention and telephone call contact attempts at 3-month, 6-month, and 12-month telephone follow-up visits among eligible participants.

3-month retention among eligible participants, n (%) *	59 of 60 (98)
3-mo call contact attempts, Median [IQR]	1[1 to 2]
Days between 3-mo telephone follow-up and 3-mo post-discharge date, Median [IQR]^a^	1[−3 to 8]
Days between 3-mo in-person follow-up and 3-mo post-discharge date, among those who consent to IPV, Median [IQR]	8[0 to 14]
6-month retention among eligible participants, n (%) *	53 of 55 (96)
6-mo call contact attempts, Median [IQR]	1[1 to 2]
Days between 6-mo telephone follow-up and 6-mo post-discharge date, Median [IQR]	3[−3 to 17]
12-month retention among eligible participants, n (%) *	31 of 32 (97)
12-mo call contact attempts, Median [IQR]	1[1 to 2]
Days between 12-mo telephone follow-up and 12-mo post-discharge date, Median [IQR]	−1[−6 to 8]

* Analyses were conducted in an ongoing study, and therefore, not all enrolled participants were yet eligible for follow-up at 6 months or 12 months. Participants who died prior to the scheduled follow-up were not considered to be eligible.

a The first three participants were excluded from the 3-month analysis, as they occurred prior to full implementation of the follow-up protocol.

**Table 4:** Stratified analyses of retention at 3-month, 6-month, and 12-month telephone follow-up visits.

Retention 3-mo, n (%) *	Younger	Older	p- value	English	Spanish	p- value	<HS Education	>=HS Education	p- value
	32 (100)	27 (96)		43 (100)	16 (94)		17 (100)	42 (98)	
3-mo call contact attempts, Median [IQR]	1[1 to 2]	1[1 to 2]	0.44	1[1 to 2]	1[1 to 2]	0.70	1[1 to 1]	1[1 to 2]	0.20
Days between 3-mo telephone follow-up and 3-mo post-discharge date, Median [IQR]^a^	1[−2 to 7]	1[−5 to 8]	0.62	2[−2 to 8]	0[−6 to 6]	0.24	0[−6 to 4]	2[−2 to 9]	0.10
Days between 3-mo in-person follow-up and 3-mo post-discharge date, among those who agreed to in-person follow-up, Median [IQR]	7[−1 to 12]	10[0 to 16]	0.30	10[0 to 16]	3[−1 to 9]	0.13	9[1 to 16]	8[−1 to 12]	0.48
**Retention 6-mo, n (%)** *	**27 (100)**	**26 (93)**		**38(100)**	**15(88)**		**17 (100)**	**36 (95)**	
6-mo call contact attempts, Median [IQR]	1[1 to 2]	1[1 to 2]	0.84	1[1 to 2]	1[1 to 2]	0.70	1[1 to 1]	1[1 to 2]	0.26
Days between 6-mo telephone follow-up and 6-mo post-discharge date, Median [IQR]	0 [−3 to 9]	4 [−3 to 28]	0.36	2 [−3 to 13]	7[−4 to 28]	0.48	1[0 to 9]	−4[3 to 27]	0.88
**Retention 12-mo, n (%)** *	**19 (100)**	**12 (92)**		**23(92)**	**8(100)**		**10 (100)**	**21 (95)**	
12-mo call contact attempts, Median [IQR]	1[1 to 3]	1[1 to 2]	0.32	1[1 to 2]	1[1 to 2]	0.83	1[1 to 2]	1[1 to 2]	1.01
Days between 12-mo telephone follow-up and 12-mo post-discharge date, Median [IQR]	0[−6 to 19]	−1[−6 to 6]	0.60	1[− 6 to 14]	−2 [−13 to 2]	0.31	−3[−7 to 7]	1[−6 to 8]	0.67

All values are calculated as median (interquartile range) or No (%). The study population median age, 61 years, was used to stratify the population into younger and older groups.

* 5 subjects died before the 3-month follow-up, 1 before the 6-month follow-up, and 4 subjects before the 12-month follow-up. They were not included in the analysis.

a The first three participants were excluded from the 3-month analysis, as they occurred prior to full implementation of the follow-up protocol.

## Abbreviations

ICU: Intensive Care Unit
ARDS: Acute Respiratory Distress Syndrome
PICS: Post Intensive Care Syndrome
CUIMC: Columbia University Irving Medical Center
6MWD: Six Minute Walk Distance
SPPB: Short Physical Performance Battery
ECMO: Extracorporeal Membrane Oxygenation
IQR: Interquartile Range
